# Statins in Orthodontics: Pharmacological Modulation of Relapse and Tooth Movement

**DOI:** 10.7759/cureus.91486

**Published:** 2025-09-02

**Authors:** Hamza Alasbily, Huda H Mohamed, Ayshan Kolemen, Ali Alkaseh, Fardous Ali Fahmi, Wael Albhbah, Sana Abdalrahman

**Affiliations:** 1 Faculty of Dentistry, University of Benghazi, Benghazi, LBY; 2 Department of Periodontology, Faculty of Dentistry, University of Benghazi, Benghazi, LBY; 3 Department of Orthodontics, Al-Mustaqbal University College of Dentistry, Hillah, IRQ; 4 Basic Medical Science, Libyan International University, Benghazi, LBY; 5 Department of Pharmacology, Faculty of Medicine, Omar Al-Mukhtar University, Al-Bayda, LBY

**Keywords:** orthodontic relapse, orthodontic tooth movement, orthodontic treatment, pleiotropic effects, statins

## Abstract

The primary aim of orthodontic treatment is to achieve optimal dental alignment and functional occlusion. However, several complications, such as root resorption, inflammatory reactions, anchorage instability, delayed tooth movement, and post-treatment relapse, may compromise long-term treatment outcomes. Statins are well-known antihyperlipidemic agents and among the most commonly prescribed drugs worldwide. Recently, statins have attracted attention as potential adjuvants in orthodontic therapy, with several preclinical findings suggesting their ability to prevent orthodontic relapse and influence the rate of tooth movement due to their anti-inflammatory, osteoanabolic, immunomodulatory, angiogenic, antioxidant, and antimicrobial properties. However, variations in statin type and dosage, route of administration, and safety profile remain considerable limitations. Furthermore, the absence of high-quality human trials and insufficient methodological rigor across existing evidence continues to restrict their translation into clinical practice. This narrative review explores the mechanisms through which statins may exert their therapeutic effects as adjuncts in orthodontic treatments, discusses the current body of evidence, and highlights the limitations currently impeding their clinical application in orthodontics.

## Introduction and background

Among dental specialties, orthodontics is concerned with the diagnosis, prevention, and treatment of malocclusions involving the teeth and jaws, aiming to enhance dental function, improve aesthetics, and support overall oral health through the use of mechanical forces, typically applied via fixed appliances or clear aligners. Although effective, orthodontic treatments present multiple mechanical as well as biological challenges that may compromise their therapeutic outcomes [1-3].

One of the frequent challenges of orthodontic treatment is orthodontic-induced inflammatory root resorption (OIIRR), which occurs in response to orthodontic forces and involves the shortening or resorption of tooth roots. The severity can range from mild to severe, with some cases posing a risk to the structural integrity of the affected teeth [3-5]. Pain and inflammation are also common complications of orthodontic treatment, particularly during the early period of orthodontic force application. These symptoms often affect mastication and speech and may reduce patient compliance. The subsidence of pain may be influenced by the type of orthodontic appliance; some studies suggest that pain resolves more quickly in patients undergoing aligner treatment, while others report a faster recovery of chewing function in those treated with fixed appliances [6,7].

Another recognized challenge of orthodontic treatment is the occurrence of periodontal complications, ranging from gingival inflammation and plaque accumulation to, in some cases, progression to periodontitis. The incidence of these issues is likely related to compromised oral hygiene around the orthodontic appliances and tends to be more severe with fixed appliance therapy [3,8]. Delayed or inefficient tooth movement also presents a significant challenge, influenced by factors such as individual biological variability, patient compliance, and the mechanics used. A key factor contributing to this delay is anchorage loss, defined as the unintended movement of anchorage units during the application of orthodontic force. Anchorage loss reduces the mechanical stability required for effective tooth movement and can manifest as a slower-than-expected response of the target teeth, despite appropriate force levels. These interconnected factors may prolong treatment duration, lead to unfavorable tooth positioning, and compromise both functional and esthetic outcomes, underscoring the essential role of precise anchorage control in successful orthodontic therapy [1,9,10].

**Figure 1:**
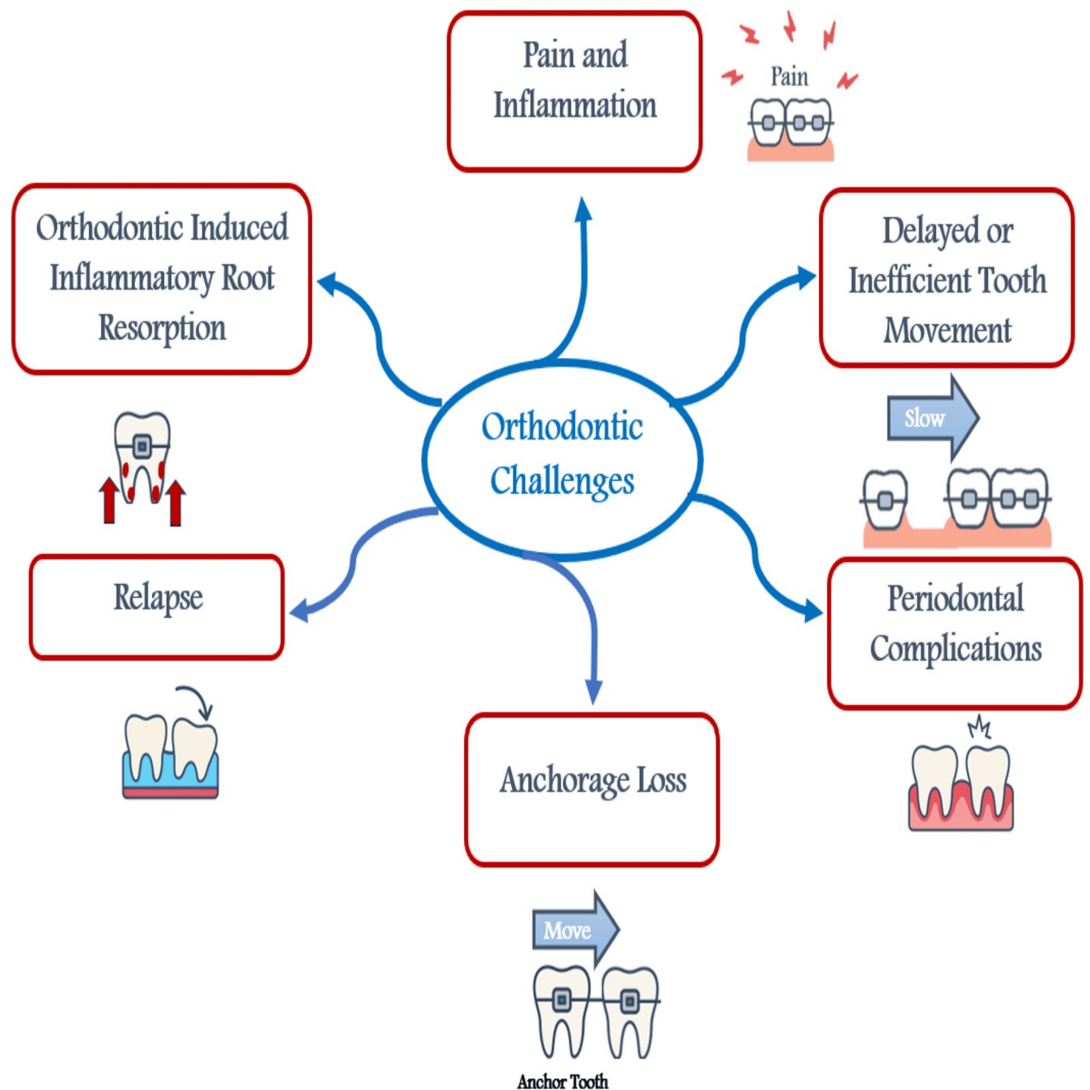
Common challenges in orthodontic treatment This information is cited from references [3-11]. Image credit: Created by the authors using Microsoft Word (Microsoft Corp., Redmond, WA, US) with icons designed using ChatGPT (OpenAI, San Francisco, US).

Moreover, orthodontic relapse, the tendency of teeth to return toward their original positions, is considered a primary challenge even after successful alignment, highlighting the importance of long-term retention strategies to maintain treatment outcomes [11]. The complexity and multifaceted nature of orthodontic challenges highlight the need for more comprehensive approaches and the development of innovative strategies to enhance treatment effectiveness and clinical outcomes. Figure 1 summarizes the common challenges encountered during orthodontic treatment.

In recent years, the pharmacological modulation of orthodontic treatment has received increasing attention, with several agents, such as bisphosphonates, metformin, parathyroid hormone analogs, and statins, being studied for their potential roles in influencing bone remodeling and inflammatory responses during the application of orthodontic forces. Statins, in particular, have attracted growing interest due to their pleiotropic effects, which may offer therapeutic advantages in addressing the common challenges associated with orthodontic treatment [12-15].

Statins are well-known antihyperlipidemic medications and are considered among the most prescribed drugs worldwide. They are primarily indicated for the management of hypercholesterolemia and the prevention of atherosclerotic cardiovascular disease (ASCVD) in diverse patient populations, including individuals with diabetes, coronary artery disease (CAD), familial hypercholesterolemia (FH), metabolic syndrome (MetS), and chronic kidney disease (CKD) [16-19].

**Figure 2:**
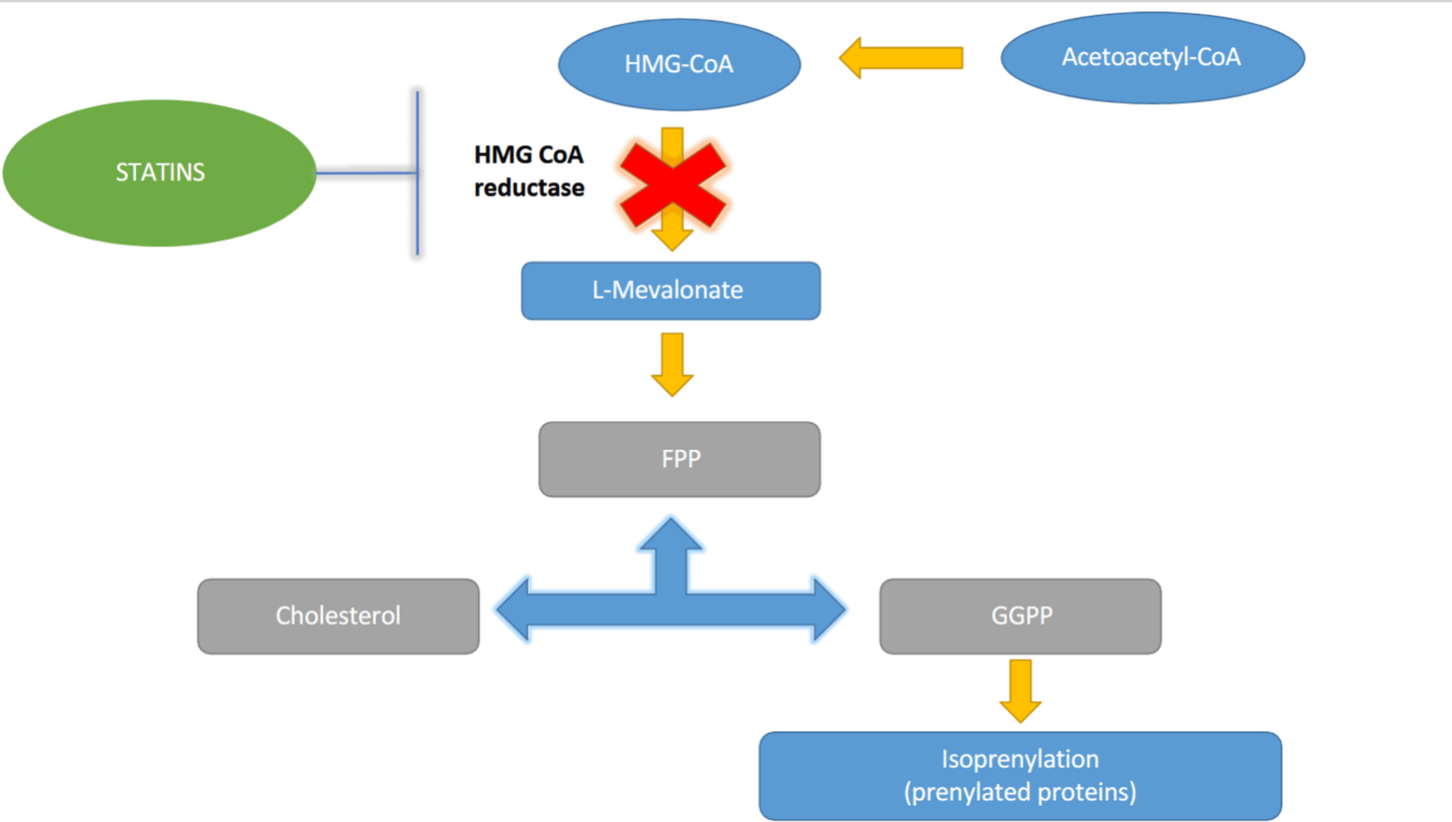
Mechanism of action of statins HMG-CoA, 3-hydroxy-3-methylglutaryl coenzyme A; Acetoacetyl-CoA, acetoacetyl coenzyme A; FPP, Farnesyl Pyrophosphate; GGPP, Geranylgeranyl Pyrophosphate. This information is cited from references [16-19]. Image credit: Created by the authors using Microsoft Word (Microsoft Corp., Redmond, WA, US)

The primary mechanism of action of statins shown in Figure 2 involves the competitive inhibition of 3-hydroxy-3-methylglutaryl coenzyme A (HMG-CoA) reductase, a rate-limiting enzyme in hepatic cholesterol biosynthesis, leading to reduced intracellular cholesterol levels and subsequently upregulating low-density lipoprotein (LDL) receptors, thereby enhancing the clearance of circulating LDL cholesterol.

Additionally, inhibition of HMG-CoA reductase disrupts the mevalonate pathway, reducing the synthesis of key isoprenoid intermediates such as farnesyl pyrophosphate (FPP) and geranylgeranyl pyrophosphate (GGPP). This effect contributes to several pleiotropic actions of statins beyond lipid regulation [16-19].

**Figure 3:**
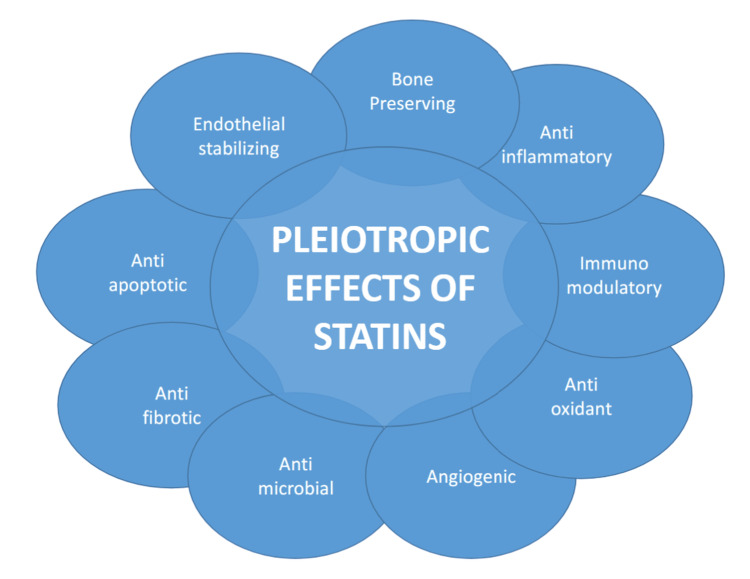
Pleiotropic effects of statins Image credit: Created by the authors using Microsoft Word (Microsoft Corp., Redmond, WA, US)

Several statins are clinically available, including simvastatin, atorvastatin, fluvastatin, rosuvastatin, pravastatin, pitavastatin, and lovastatin, each exhibiting unique pharmacokinetic and pharmacodynamic properties. The lipophilic statins (e.g., atorvastatin, simvastatin) undergo extensive first-pass metabolism and therefore get distributed more readily into extrahepatic tissues. Conversely, the hydrophilic statins (e.g., rosuvastatin, pravastatin) exhibit greater hepatoselectivity and limited penetration into peripheral tissues. Additionally, genetic polymorphisms may alter both the therapeutic response to statins and the likelihood of adverse effects, including myopathy, elevated liver enzymes, and a modest increase in the risk of developing new-onset diabetes mellitus [16-19]. The pleiotropic effects of statins cover a broad spectrum of actions, including endothelial-stabilizing, bone-preserving, anti-inflammatory, immunomodulatory, antioxidant, pro-angiogenic, antimicrobial, anti-fibrotic, and anti-apoptotic properties, as illustrated in Figure 3.

Owing to these diverse actions, the potential benefits of statins have been investigated across multiple disciplines, such as ophthalmology, nephrology, dermatology, immunology, and infectious diseases [16,20,21].

In dentistry, statins have shown the ability to modulate several biological pathways involved in the maintenance of oral health and the pathogenesis of oral diseases. For instance, in periodontology, statins have demonstrated beneficial outcomes in periodontal disease prevention, alveolar bone preservation, and as adjuncts by exerting positive effects on surgical and non-surgical periodontal treatments [22-24]. Furthermore, emerging evidence supports their potential benefits across other dental specialties, such as endodontics, implantology, and oral and maxillofacial surgery [25-28]. Regarding orthodontics, the anti-inflammatory, osteopromotive, periodontal-stabilizing, and immunoregulatory effects of statins offer a strong rationale for their use as adjunctive agents. While current evidence is limited and mainly preclinical, further comprehensive research is needed to clarify their roles and potential benefits in the orthodontic context. This narrative review explores the mechanisms through which statins may exert their therapeutic effects as adjuncts in orthodontic treatments, discusses the existing evidence, and identifies the limitations of their potential role in orthodontics.

Research methodology

In conducting this narrative review, an extensive search was performed across major scientific databases, including PubMed, Scopus, Web of Science, Google Scholar, and Directory of Open Access Journals (DOAJ), as well as publishing platforms such as ScienceDirect, Wiley Online Library, SpringerLink, Taylor & Francis Online, and Multidisciplinary Digital Publishing Institute (MDPI). The search focused primarily on peer-reviewed, English-language articles published between 2020 and July 2025, a period chosen to emphasize the most recent and clinically relevant evidence on the role of statins in orthodontics. The search strategy combined Medical Subject Headings (MeSH) and free-text keywords, including: “Statins” [MeSH], “Orthodontics” [MeSH], “orthodontic tooth movement,” “orthodontic relapse,” “root resorption,” “alveolar bone remodeling,” and “pleiotropic effects.” Boolean operators (AND/OR) were used to refine results. Additional references were identified by manually screening the bibliographies of relevant articles.

## Review

Proposed mechanisms of statins in orthodontics

Anti-Inflammatory Actions 

The anti-inflammatory properties of statins are primarily attributed to their modulation of key inflammatory signaling pathways, underscoring their potential as adjunctive agents for regulating biological responses.

As the inhibition of HMG-CoA reductase disrupts the mevalonate pathway, the synthesis of isoprenoid intermediates, such as FPP and GGPP, is decreased. These molecules are essential for the prenylation and activation of small guanosine triphosphate (GTP)-binding proteins, including Ras homolog family member (Rho), Ras-related C3 botulinum toxin substrate (Rac), and rat sarcoma (Ras) GTPases, which initiate pro-inflammatory signaling cascades. By targeting these intracellular pathways, statins reduce the nuclear translocation and transcriptional activity of nuclear factor-kappa B (NF-κB), resulting in significant suppression of key cytokines, such as interleukin-1β (IL-1β), interleukin-6 (IL-6), and tumor necrosis factor-alpha (TNF-α), thereby attenuating the inflammatory response. Simultaneously, statins enhance the expression of IL-10, a potent anti-inflammatory cytokine that counteracts pro-inflammatory mediators and supports immunological homeostasis. This effect is partly attributed to NF-κB inhibition and may also involve activation of peroxisome proliferator-activated receptors (PPARs), particularly PPAR-γ, which regulate anti-inflammatory transcriptional networks [16,29-31].

Macrophage polarization provides an additional anti-inflammatory mechanism whereby statins facilitate a shift from classically activated M1 macrophages, characterized by pro-inflammatory and osteolytic activity, toward alternatively activated M2 macrophages, which secrete cytokines such as IL-10 and transforming growth factor-beta (TGF-β), both essential for the resolution of inflammation and the promotion of tissue remodeling. This phenotypic reprogramming is proposed to foster a reparative microenvironment that may support balanced alveolar bone turnover during orthodontic tooth movement (OTM) [29,31,32]. Moreover, statins suppress the expression and enzymatic activity of matrix metalloproteinases (MMPs), particularly MMP-2 and MMP-9. These proteolytic enzymes, frequently elevated during inflammatory conditions, contribute to extracellular matrix degradation and have been implicated in OIIRR. Statins downregulate MMP transcription through inhibition of both NF-κB and activator protein-1 (AP-1), which may aid in preserving periodontal ligament integrity and limiting soft tissue breakdown [30,31].

In addition to the previously mentioned mechanisms, the antioxidant properties of statins further support their anti-inflammatory role by reducing the generation of reactive oxygen species (ROS), which are critical amplifiers of local inflammation and mediators of oxidative damage to alveolar and periodontal structures. Statins exert this effect by inhibiting nicotinamide adenine dinucleotide phosphate (NADPH) oxidase activity and enhancing the expression of endogenous antioxidant enzymes, including superoxide dismutase (SOD), thereby maintaining redox homeostasis within periodontal tissues [16,30,33,34].

Angiogenic Effects

Statins promote angiogenesis through multiple interrelated pathways that enhance endothelial function, vascular remodeling, and tissue repair, all of which could be critical for supporting the dynamic changes during OTM.

A primary mechanism for the angiogenic properties of the statins is the upregulation of vascular endothelial growth factor (VEGF), a key regulator of angiogenesis. Statins enhance the expression of VEGF by activating the phosphatidylinositol 3-kinase (PI3K)/protein kinase B (Akt) signaling pathway, which promotes endothelial cell proliferation, directed migration, cytoskeletal reorganization, and the formation of capillary-like structures. The activation of the PI3K/Akt pathway also supports the endothelial cell survival via suppressing pro-apoptotic signals, thereby maintaining vascular integrity in response to mechanical stress caused by orthodontic forces. Additionally, statin-induced angiogenesis involves the activation of endothelial nitric oxide synthase (eNOS), which increases the availability of nitric oxide (NO). eNOS activation is mediated through PI3K/Akt-dependent phosphorylation and the inhibition of caveolin-1, an endogenous negative regulator of eNOS. NO serves as a key angiogenic mediator by inducing vasodilation, increasing vascular permeability, and promoting endothelial cell adhesion and migration. Elevated NO levels not only enhance angiogenic signaling but also potentially exert cytoprotective effects that stabilize the microvascular environment during orthodontic-induced inflammation and tissue remodeling [35-37].

Besides their local effects on endothelial cells, statins promote the mobilization of endothelial progenitor cells (EPCs) from bone marrow niches into the peripheral circulation. EPCs contribute to postnatal vasculogenesis by homing in on sites of vascular remodeling, differentiating into mature endothelial cells, and incorporating themselves into the neovasculature. Statins facilitate EPC mobilization through upregulation of stromal cell-derived factor-1 (SDF-1)/C-X-C chemokine receptor type 4 (CXCR4) signaling and by enhancing VEGF and eNOS expression within the bone marrow microenvironment. In the context of orthodontics, EPC-mediated revascularization of the periodontal ligament and alveolar bone may support tissue regeneration and biomechanical adaptation to applied orthodontic forces [35,37,38]. Furthermore, the antioxidant properties of the statins also contribute to endothelial preservation by reducing oxidative stress, thereby sustaining an angiogenic microenvironment and preventing vascular dysfunction during active tissue remodeling [33-35].

Promotion of Osteoblast Differentiation and Bone Regeneration

Statins exert multifaceted effects on bone remodeling by promoting osteoblast differentiation, inhibiting osteoclast activity, and modulating inflammatory and angiogenic pathways critical for alveolar bone homeostasis during OTM.

Statins stimulate osteoblastic differentiation, matrix synthesis, and mineralization primarily by upregulating bone morphogenetic protein-2 (BMP-2), a pivotal osteoinductive factor that directs mesenchymal stem cells toward an osteoblastic lineage. This induction activates key intracellular signaling cascades, including extracellular signal-regulated kinase (ERK), p38 mitogen-activated protein kinase (MAPK), and PI3K/Akt. Subsequently, the expression of key osteogenic transcription factors, including Runt-related transcription factor 2 (Runx2) and Osterix, is increased, which controls genes involved in osteoblast function and matrix synthesis. As a result, statins increase the expression of osteoblast-specific markers, including alkaline phosphatase (ALP), osteocalcin, and type I collagen, that are crucial for extracellular matrix assembly and mineral deposition. Collectively, these molecular events may contribute to alveolar bone formation, potentially improving the long-term stability of orthodontic outcomes. In parallel, statins suppress osteoclastogenesis, thereby promoting a remodeling environment that favors bone formation over resorption [39-43]. This dual action, enhancing bone formation while inhibiting resorption, results in increased alveolar bone volume, which may reduce the risk of orthodontic relapse and shorten the retention period required after treatment. Importantly, statins appear to protect against OIIRR by diminishing osteoclast-like activity at the root surface. By inhibiting the mevalonate pathway, statins suppress the expression of MMPs and pro-resorptive cytokines, thereby preserving the structural integrity of the root cementum [39-43].

Beyond their direct osteogenic effects, the immunomodulatory and angiogenic properties of statins further facilitate bone regeneration. By inhibiting the NF-κB signaling pathway and suppressing pro-inflammatory cytokines such asTNF-α and IL-6, statins mitigate inflammation-driven impairment of osteoblast differentiation. Simultaneously, the upregulation of anti-inflammatory cytokines, including IL-10, fosters a regenerative microenvironment that sustains osteoblastic function under mechanical and oxidative stress [16,29-31]. Moreover, the promotion of angiogenesis by the statins enhances vascularization, thereby ensuring adequate nutrient delivery and the recruitment of osteoprogenitor cells, processes essential for the coordination of osteogenesis and angiogenesis during bone remodeling [35-37].

Inhibition of Osteoclast Differentiation

Statins exert inhibitory effects on osteoclast-mediated bone resorption by targeting key molecular pathways that regulate osteoclast differentiation and function, thereby contributing to the preservation of alveolar bone during orthodontic treatment.

The primary anti-resorptive effect of statins results from inhibition of the mevalonate pathway, disrupting the synthesis of isoprenoid intermediates such as FPP and GGPP. These intermediates are essential for osteoclast functions, including cytoskeletal organization, actin ring formation, vesicular trafficking, and ruffled border development required for bone resorption. Their depletion impairs osteoclast polarization and reduces their resorptive activity [39,43-45].

Concurrently, statins modulate the receptor activator of nuclear factor-kappa B ligand (RANKL)/osteoprotegerin (OPG) signaling axis, a key regulator of osteoclast differentiation and activation. Statins increase the expression of OPG, a soluble decoy receptor that binds RANKL, thereby preventing its interaction with RANK on osteoclast precursors. This shift in the RANKL/OPG ratio inhibits osteoclast recruitment and differentiation, thereby reducing osteoclastogenesis at the sites of bone remodeling [39,45].

Additionally, statins downregulate essential transcription factors involved in osteoclast development, including cellular Finkel-Biskis-Jinkins murine osteosarcoma viral oncogene homolog (c-Fos) and nuclear factor of activated T-cells cytoplasmic 1 (NFATc1). These factors, activated downstream of RANK through TNF receptor-associated factor 6 (TRAF6) and NF-κB signaling, are necessary for the expression of osteoclast-specific genes such as tartrate-resistant acid phosphatase (TRAP), cathepsin K, and the calcitonin receptor. Statin-mediated suppression of these pathways interferes with the transcriptional program necessary for osteoclast formation and function [39,45].

Support of Periodontal Health

Statins play a significant role in the preservation and enhancement of periodontal health through various mechanisms, many of which, such as their anti-inflammatory, antioxidant, and bone-preserving properties, have been discussed earlier. Collectively, these effects support favorable outcomes during orthodontic treatment by maintaining the structural and functional integrity of periodontal tissues. Among these mechanisms, the antimicrobial actions of statins deserve particular emphasis due to their increasing relevance in both periodontal and orthodontic contexts.

Statins have demonstrated efficacy in inhibiting the growth, adhesion, and biofilm formation of key periodontal pathogens, including Porphyromonas gingivalis, Aggregatibacter actinomycetemcomitans, Fusobacterium nucleatum, and Tannerella forsythia, organisms that play central roles in the pathogenesis of periodontitis and compromise periodontal stability during OTM. Through both systemic and local delivery, statins have shown promise in managing and preventing periodontal diseases by modulating the subgingival microbiota and preserving tissue integrity. This microbial modulation promotes a more balanced and less pathogenic oral environment [46,47], which correlates with a reduction in plaque accumulation and gingival inflammation, along with improvements in clinical parameters such as probing depth (PD), bleeding on probing (BOP), and gingival index (GI) [48-50]. These microbiological benefits are particularly valuable during orthodontic therapy, where fixed appliances increase plaque retention and the risk of soft tissue complications.

Moreover, the established anti-inflammatory effects of statins help modulate the host immune response, reducing tissue degradation and promoting periodontal healing. Their antioxidant properties also play a supportive role by lowering oxidative stress through the increased activity of endogenous enzymes such as SOD and glutathione peroxidase (GPx), which help maintain redox balance within periodontal tissues. Although the bone-preserving actions of statins, such as the stimulation of osteoblast activity, induction of BMP-2, and VEGF-mediated angiogenesis, have been discussed previously, it is important to highlight that these effects synergize with microbial suppression and immune modulation to enhance periodontal resilience [16,29,34,35]. Through these diverse mechanisms, statins underscore their therapeutic potential as adjunctive agents in preserving periodontal health and optimizing tissue responses throughout orthodontic treatment. Table 1 summarizes the proposed mechanisms and related pharmacological actions of statin use in orthodontics.

**Table 1:** Proposed mechanisms of statins in orthodontics NF-κB , Nuclear Factor kappa-light-chain-enhancer of activated B cells; IL-1β, interleukin-1β; IL-6, interleukin-6; TNF-α, Tumor Necrosis Factor alpha; IL-10, interleukin-10; PPAR-γ, Peroxisome Proliferator-Activated Receptor gamma; MMPs, Matrix Metalloproteinases; ROS, Reactive Oxygen Species; PI3K, Phosphoinositide 3-kinase; Akt, Protein Kinase B; VEGF, Vascular Endothelial Growth Factor; eNOS, Endothelial Nitric Oxide Synthase; EPCs, Endothelial Progenitor Cells; Runx2, Runt-related transcription factor 2; RANK, Receptor Activator of Nuclear Factor κB; OPG, Osteoprotegerin; c-Fos, Cellular oncogene Fos; NFATc1, Nuclear Factor of Activated T-cells, cytoplasmic 1.

Pharmacological actions	Mechanism	References
Anti-inflammatory	• ↓NF-κB → ↓IL-6, IL-1β, TNF-α, ↑IL-10 • PPAR-γ → ↑IL-10 • Macrophage polarization M1 → M2 • ↓MMPs • ↓ROS	[16,29-34]
Angiogenic	• PI3K/Akt pathway activation →↑VEGF, eNOS • EPCs mobilization • ↓ROS → endothelial stabilization	[33-38]
Promotion of osteoblast differentiation/bone regeneration	• ↑Runx2, Osterix • ↓IL-6, TNF-α, IL-1β • Angiogenesis support	[16,29,35-37,39-43]
Osteoclast inhibition	• ↓Osteoclast polarization & resorptive activity • Disruption of RANK–OPG axis • ↓c-Fos, NFATc1	[39,43-45]
Support of periodontal health	• Antibacterial actions • Anti-inflammatory effects • Bone preservation effects • Antioxidant effects	[16,29,34,35,46-50]

Evidence on statins in orthodontics

Statins and Orthodontic Relapse

In multiple preclinical studies, experimental trials, and systematic reviews, statins have attracted growing research interest for their potential to prevent orthodontic relapse, largely through modulation of bone remodeling processes.

The effect of various pharmacological agents on orthodontic relapse after treatment was assessed by a systematic review of animal studies by Veginadu et al. The search identified seven eligible studies, most of which were marked by unclear risk of bias and generally low methodological quality. Among the agents evaluated, statins showed variable results: atorvastatin tended to reduce relapse, whereas findings for simvastatin were inconsistent. Importantly, the data suggest a dose-dependent response: systemic low-dose simvastatin was associated with relapse reduction, while systemic high-dose or locally administered low-dose simvastatin had fewer clear effects. These variations may be attributed to differences in bone cell sensitivity to drug concentration, delivery methods, animal models, and treatment duration. Although statins appear promising as adjuncts to reduce post-orthodontic relapse, further rigorous and high-quality studies are necessary to confirm their efficacy and inform clinical practice [51].

Another systematic review conducted by Afshari et al. assessed animal studies evaluating the role of statins in preventing orthodontic relapse. Seven trials were ultimately included. While some studies have reported the ability of statins to effectively reduce relapse, others have found no statistically significant differences. Specifically, four studies observed a decrease in relapse rates, whereas three reported no notable changes. Given these mixed results and the methodological variability among the included studies, the authors concluded that current animal evidence remains insufficient to confirm the efficacy of statins in preventing orthodontic relapse. Consequently, due to the observed discrepancies in outcomes and study designs, statins cannot yet be recommended for clinical orthodontic use in this context until robust, high-quality studies are conducted [52].

A promising case-control study by Abu-Bakr et al. examined the effect of oral atorvastatin on orthodontic relapse using an animal model of 16 healthy male New Zealand white rabbits with fully developed dentition. Tooth movement was induced by placing a closed-coil Ni-Ti Spring between the lower incisors and first premolars. After three weeks, the appliance was removed and baseline measurements recorded. The rabbits were then randomly assigned to two groups: the intervention group received 20 mg/kg of atorvastatin calcium suspended in distilled water via oral gavage once daily for 21 days, while the control group received an equivalent volume of phosphate-buffered saline. After the treatment period, relapse was evaluated, and one animal from each group was selected for histological examination. Histomorphometric analyses included counts of osteoblasts and osteoclasts, cortical bone thickness, trabecular architecture, bone density, and vascularization. The results indicated a greater relapse percentage in the control group compared to the atorvastatin-treated group, suggesting a protective effect of statin therapy. While the mean osteoblast count was slightly higher in controls (not statistically significant), the osteoclast count was significantly lower in the atorvastatin group. Interestingly, bone density was reduced in the atorvastatin-treated group, suggesting the need for further investigation. Overall, the findings support a potential role for atorvastatin in mitigating post-orthodontic relapse through modulation of bone remodeling processes [53].

Similarly, a preclinical study by Nascimento et al. investigated the effects of systemic simvastatin on bone remodeling within the midpalatal suture following rapid maxillary expansion (RME), offering additional mechanistic insight into the potential of statins in enhancing post-orthodontic stability. In this controlled study, 15 Wistar rats were divided into three groups: untreated controls, RME with distilled water, and RME combined with simvastatin treatment (5 mg/kg/day for 20 days). After five days of active expansion, the simvastatin group demonstrated significantly greater bone regeneration at the expansion site. Micro-computed tomography (µCT) analysis showed increased bone volume fraction (BV/TV), cortical thickness (Ct.Th), and cortical area (Ct.Ar), accompanied by reduced void spaces and improved linear remodeling metrics at multiple anatomical locations. Histological and histomorphometric evaluations corroborated these findings, demonstrating increased osteoblastic activity and decreased osteoclastic resorption in the statin group. Although this study did not directly evaluate relapse of tooth movement, it emphasizes the role of midpalatal suture remodeling in maintaining long-term skeletal stability after expansion. Therefore, simvastatin’s osteoinductive and antiresorptive actions may indirectly contribute to relapse prevention, supporting its use as an adjunct in orthodontic retention strategies [54].

In addition, various local delivery systems have been explored to enhance statin efficacy and site-specific targeting. Rosyida et al. assessed the use of a gelatin-simvastatin hydrogel to improve orthodontic retention in a rabbit model. Twenty-four rabbits underwent tooth movement followed by a two-week stabilization period, during which the treatment group received the simvastatin-loaded hydrogel, while the control group was administered blank hydrogel. Upon removal of the orthodontic appliances, relapse was initiated, and the extent of tooth movement reversal was measured using digital calipers. Molecular analysis of bone remodeling markers was performed using an enzyme-linked immunosorbent assay (ELISA) to quantify levels of OPG and RANKL. The intervention significantly reduced relapse percentage and RANKL expression, while increasing OPG and the OPG/RANKL ratio, reflecting enhanced osteogenesis and suppressed bone resorption. These results suggest that localized simvastatin delivery facilitates bone remodeling and may offer a minimally invasive adjunctive strategy to improve post-orthodontic stability [55].

Alhasyimi et al. demonstrated that topical application of a nanoemulsion combining statin with carbonated hydroxyapatite (CHA-statin) effectively prevents orthodontic relapse in rats by promoting bone formation and inhibiting bone resorption. Forty-eight rats were allocated into four groups: control, CHA, statin, and CHA-statin. OTM was induced using a 30 g mesial force for seven days via a closed-coil spring. Following device removal, treatments were applied intrasulcularly every three days for seven days to maintain tooth position during the relapse phase. Immunohistochemical analysis revealed that the CHA-statin group exhibited significantly fewer tartrate-resistant acid phosphatase (Acp5)-positive osteoclasts on days seven and 14 (p<0.05), along with increased Runx2 expression on days one, seven, and 14 (p<0.05), indicating enhanced bone remodeling. These effects were accompanied by elevated calcium and phosphate levels, essential for new bone formation and inhibition of osteoclastic activity associated with relapse [56].

Yaseen et al. also evaluate the effect of local rosuvastatin injection delivered via a hyaluronan hydrogel (HAH) on alveolar bone regeneration following OTM. Thirty albino rabbits were randomly assigned to two groups: one received 200 μL of phosphate-buffered saline (control), while the other was administered 200 μL of rosuvastatin/HAH locally on the first day of retention after distal movement of the lower incisors. Histological analyses quantified osteoblasts, osteoclasts, and blood vessels, whereas immunohistochemical assessments measured bone alkaline phosphatase (BAP) and TRAP 5b expression at coronal and apical sites on days 0, 10, and 21 post the injections. Statistical analysis revealed significant differences (p≤0.05) between groups. The rosuvastatin/HAH group showed markedly increased osteoblast counts and BAP expression on days 0 and 10, along with decreased osteoclast counts and TRAP 5b expression. Additionally, blood vessel counts were significantly higher on day 10 at both coronal and apical levels. These findings suggest that local rosuvastatin/HAH injection promotes alveolar bone regeneration by enhancing osteoblastic activity and angiogenesis while suppressing osteoclastic resorption, supporting its potential as an adjunctive approach to improve bone remodeling and orthodontic stability [57].

Additionally, Liu et al. demonstrated that encapsulating simvastatin within exosomes derived from periodontal ligament stem cells (PDLSCs) significantly improves its solubility and enhances inhibition of relapse following OTM in a rat model. PDLSCs and their exosomes (PDLSCs-Exo) were isolated and characterized, then co-incubated with simvastatin to produce exosomal simvastatin. During the relapse phase, local alveolar bone injections of simvastatin, PDLSCs-Exo, or exosomal simvastatin were administered. The results showed that exosomal simvastatin markedly reduced relapse rate and distance compared to controls, outperforming free simvastatin and PDLSCs-Exo alone. Molecular and histological analyses revealed increased expression of osteogenic genes and proteins alongside decreased bone resorption in the exosomal simvastatin group. These findings suggest that exosome-mediated delivery of simvastatin represents a promising strategy to enhance post-orthodontic stability through improved bone remodeling [58].

Overall, the previous preclinical evidence suggests that statins may reduce orthodontic relapse by enhancing osteogenesis and inhibiting bone resorption through multiple molecular pathways. The effectiveness of statins appears to depend on factors such as the specific statin used, dosage, formulation, and route of administration. Although the existing evidence is generally rated as low-to-moderate quality, this is primarily due to variability between studies, unclear risk of bias, and inconsistent outcome measures.

Statins and OTM

The ability of statins to influence OTM has gained increasing attention, with both systemic and local administration investigated in preclinical studies and systematic reviews. These findings carry important clinical implications, especially regarding anchorage preservation and the optimization of treatment duration.

In a systematic review involving 27 animal studies, Makrygiannakis et al. evaluated commonly prescribed medications, including vitamin C, pantoprazole, calcium compounds, strontium ranelate, propranolol, losartan, famotidine, cetirizine, metformin, and statins such as simvastatin and atorvastatin, for their effects on the rate of OTM. The findings indicated that simvastatin and atorvastatin generally reduced the rate of OTM by modulating bone remodeling. This effect appeared dose-dependent, with the lipophilic simvastatin exerting a more pronounced influence on bone turnover than the hydrophilic atorvastatin. Although the overall quality of evidence was low, limiting the strength of clinical recommendations, the authors emphasized the need to consider the medication profiles of the patients and potential drug-related effects when planning orthodontic treatment [59].

Supporting these findings, Dolci et al. evaluated whether atorvastatin modulates OTM by inhibiting osteoclast activity and assessed its effects on long-bone turnover and endochondral ossification in rats. Two groups of twenty-four male rats were used: one received saline, while the other was administered oral atorvastatin (15 mg/kg/day) starting two weeks before OTM induction. Orthodontic appliances were applied to move the first molar, and tooth movement was measured at seven, 14, and 21 days. Histological analysis assessed osteoclast activity and bone morphology in both the jaw and femur. Atorvastatin-treated rats exhibited significantly reduced tooth movement and osteoclast numbers (p<0.05), indicating suppressed bone resorption as the underlying mechanism. Importantly, atorvastatin did not affect femoral bone development, suggesting its inhibitory effects on OTM occur without adverse impacts on systemic bone health [60].

Similarly, AlSwafeeri et al. investigated the effect of locally injected simvastatin on OTM using a split-mouth design in 10 white New Zealand rabbits. One side of the mandible received simvastatin, while the contralateral side was treated with a control solution. Orthodontic force was applied via nickel-titanium coil springs over 21 days, and tooth movement was measured using three-dimensional dental models. Postmortem histomorphometric analysis of alveolar bone assessed bone remodeling. The results showed a 39.86 ± 22.6% reduction in tooth movement on the simvastatin-treated side, which correlated with fewer osteoclasts and decreased active bone resorption. These findings indicate that local simvastatin delivery can inhibit bone remodeling in response to orthodontic force, presenting a potential approach for anchorage preservation by limiting undesired tooth movement [61].

The local delivery of simvastatin has also demonstrated the ability to maintain tooth anchorage during mechanical tooth movement, as shown by Xu et al., who investigated its effects on PDLCs. Their study revealed that a low dose of simvastatin (0.05 μM) promoted osteogenic differentiation of PDLCs via activation of AMPK, a critical mediator protecting tissues from inflammation induced by mechanical stress. In a rodent model, simvastatin-induced activation of AMP-activated protein kinase (AMPK) significantly reduced bone resorption and decreased inflammatory mediators associated with OTM. These findings suggest that simvastatin may support anchorage preservation by reducing inflammation and promoting bone formation through the AMPK/MAPK/NF-κB signaling pathway. Nonetheless, the authors stressed the need for further studies to clarify the complex interactions between mechanical forces and osteoclast-mediated bone remodeling during orthodontic treatment [62].

While promising results have been shown in earlier studies, several systematic reviews have pointed out methodological inconsistencies and the generally low quality of available evidence. In a systematic review by Kommuri et al., which evaluates the impact of statins on OTM, encompassing studies published through December 2018, the review included nine studies, one clinical and eight animal-based, with six employing simvastatin and three with atorvastatin. While seven studies (six experimental and one clinical) reported a reduction in OTM with statin use, two experimental studies found no such effect. The authors noted considerable methodological variability and a high risk of bias in 90% of the included studies. Differences in animal models, statin types, dosages, and administration routes contributed to inconsistent findings. Consequently, the effect of statins on OTM remains inconclusive and warrants further investigation [63].

Similarly, Afshari et al. performed a meta-analysis evaluating the effect of statins on OTM in animals undergoing orthodontic treatment. Their systematic search, conducted through January 2020, identified three randomized animal trials meeting inclusion criteria. Each study presented at least one domain with a high risk of bias. The pooled analysis revealed a small, non-significant reduction in tooth movement associated with statin administration, likely mediated by suppression of bone resorption and inflammatory markers. However, the evidence was limited by small sample sizes, methodological heterogeneity, and the lack of human trials. The authors concluded that current animal-based evidence remains insufficient to draw definitive conclusions regarding the impact of statins on OTM [64].

Aiming to address the clinical implications of delayed orthodontic movement in patients receiving simvastatin, Fernandes et al. investigated whether photobiomodulation (PBM) could mitigate this effect by modulating bone metabolism. In their experimental study involving 56 male Wistar rats, the animals were assigned to four groups: G1-no orthodontic movement or simvastatin (n=8); G2-orthodontic movement with simvastatin (2.5 mg/kg) (n=16); G3-orthodontic movement with saline and low-level laser (808 nm, 1 J/point) (n=16); and G4-orthodontic movement with simvastatin and PBM (n=16). Simvastatin was administered for 14 days, and orthodontic movement was induced using mini-implants over seven or 14 days. PBM was applied every 48 hours. The results showed that simvastatin significantly slowed tooth movement; nonetheless, this inhibitory effect was counteracted when combined with PBM, which promoted greater tooth displacement. Supporting these findings, biochemical analyses revealed increased bone volume and elevated MMP-2 expression in the PBM-treated groups. The authors concluded that PBM could serve as an effective adjunct to counteract simvastatin-induced delays in orthodontic movement, potentially normalizing treatment durations. Interestingly, the delayed movement observed with simvastatin may also present a clinical advantage in specific cases, such as enhanced anchorage control, highlighting a potential therapeutic benefit worth further exploration in clinical practice and narrative reviews [65].

In summary, preclinical evidence largely indicated that statins, particularly simvastatin and atorvastatin, could reduce OTM primarily by inhibiting osteoclast activity and modulating inflammatory pathways. These actions suggest potential benefits for anchorage reinforcement or in clinical contexts where slower tooth movement is desired. However, the current evidence is limited by methodological inconsistencies, small sample sizes, and the absence of robust human trials. Consequently, clinical application remains preliminary, underscoring the need for well-designed studies to more clearly establish the therapeutic potential and safety of statins in orthodontic settings.

Comparative safety and efficacy of statins with conventional orthodontic treatments

In orthodontics, nonsteroidal anti-inflammatory drugs (NSAIDs), corticosteroids, and chlorhexidine are commonly used as adjunctive therapies; however, despite their clinical benefits, each presents specific limitations that may affect treatment outcomes.

The use of NSAIDs (e.g., paracetamol, ibuprofen) is common in orthodontic practice to alleviate pain and inflammation. By inhibiting cyclooxygenase (COX) enzymes, NSAIDs reduce prostaglandin synthesis and moderate the inflammatory response. However, the use of these agents may affect OTM through the alteration of bone remodeling, with outcomes depending on the type of NSAID, dosage, and duration of therapy. Patient-specific factors must also be taken into account, including contraindications in individuals with conditions such as asthma. Moreover, gastrointestinal side effects, most notably gastritis, remain among the most frequently reported adverse events [66-70].

Corticosteroids (e.g., dexamethasone gel) are commonly used in orthodontic practice to relieve inflammation linked with the treatment. Through their anti-inflammatory properties, which are linked to the downregulation of pro-inflammatory cytokines and the inhibition of leukocyte migration, corticosteroids reduce tissue swelling and inflammatory reactions. However, prolonged or excessive use may cause local adverse effects, including mucosal thinning, delayed healing of mucosa and bone, increased susceptibility to secondary infections such as candidiasis, localized tissue atrophy, and potential alterations of the normal oral microbiota. Additionally, repeated application can result in taste disturbances and mucosal irritation. Although systemic absorption is minimal with local administration, occasional systemic corticosteroid-related side effects may still occur [71-73].

Chlorhexidine, widely recognized as the standard antimicrobial agent in orthodontic care, effectively controls plaque accumulation and prevents gingival inflammation and periodontal infections during treatment. However, its clinical use is limited by several adverse effects, including mucosal irritation and ulceration, extrinsic staining of teeth and tongue, altered taste sensation (dysgeusia), increased calculus formation, and mucosal desquamation. Prolonged or excessive application may also exert cytotoxic effects on oral fibroblasts and epithelial cells. Therefore, these considerations underscore the importance of limiting use to short durations to balance antimicrobial efficacy with a minimized risk of adverse effects [74-76].

Compared with conventional orthodontic adjuncts, statins offer multiple actions that together surpass the effects of individual agents, combining anti-inflammatory, antimicrobial, and bone anabolic properties. Unlike NSAIDs and corticosteroids, which mainly target inflammation, statins provide a more comprehensive approach to enhancing periodontal health and bone remodeling. Their local delivery may allow targeted effects with minimal systemic absorption, reducing risks commonly associated with corticosteroids and NSAIDs. Additionally, unlike chlorhexidine, statins are not known to cause mucosal irritation or staining, making them a potentially safer and more effective adjunct in orthodontic treatment [16,19,50].

Current limitations of statin use in orthodontic treatment

Optimal therapeutic efficacy of statins as successful adjuncts in orthodontics is limited by variability in statin type, formulation, dosing, and delivery methods, all of which contribute to inconsistent outcomes. Moreover, much of the current evidence is derived from preclinical studies, with a notable lack of well-designed human trials to confirm both safety and effectiveness. In addition, potential systemic side effects, patient-specific variability, and possible interactions with other medications further complicate their clinical use [16,19,51,52,63].

## Conclusions

Statins have emerged as promising adjuncts in orthodontic treatment owing to their pleiotropic properties, including their ability to modulate inflammation, promote angiogenesis, support bone remodeling, and exert antioxidant, antimicrobial, and periodontal-protective actions. These aspects position them as potential agents for minimizing common orthodontic-related complications.

Nevertheless, despite encouraging findings from in vitro and animal studies, clinical translation remains limited. Differences in statin type, dosing regimens, administration routes, and unresolved safety issues remain major challenges. In addition, the current absence of high-quality, well-controlled clinical trials limits the ability to draw firm conclusions about their therapeutic safety, efficacy, and appropriate use in orthodontic settings. Careful clinical evaluation and well-designed human studies are essential before routine use in orthodontic practice. While early findings suggest statins may hold promise as adjunctive agents in orthodontics, their clinical value must be confirmed through more comprehensive and methodologically sound human studies.
